# Current Rural Drug Use in the US Midwest

**Published:** 2016-08-17

**Authors:** Kirk Dombrowski, Devan Crawford, Bilal Khan, Kimberly Tyler

**Affiliations:** Department of Sociology, Oldfather 708, University of Nebraska-Lincoln, Lincoln NE 68588, USA

**Keywords:** Rural drug use, Methamphetamine, HIV, Opioids, Addiction

## Abstract

The nature and challenge of illicit drug use in the United States continues to change rapidly, evolving in reaction to myriad social, economic, and local forces. While the use of illicit drugs affects every region of the country, most of our current information about drug use comes from large urban areas. Data on rural drug use and its harms justify greater attention. Record overdose rates, unexpected outbreaks of HIV, and a dearth of treatment facilities point to a rapidly worsening health situation. While health sciences have made considerable progress in understanding the etiology of drug use and uncovering the link between drug use and its myriad associated harms, this promising scientific news has not always translated to better health outcomes. The scope of the problem in the Central Plains of the US is growing, and can be estimated from available sources. Clear remedies for this rising level of abuse are available, but few have been implemented. Suggestions for short-term policy remedies are discussed.

## Introduction

The use of illicit drugs affects every region of the United States, but most of our information about drug use comes from large urban areas [1]. This is true despite two decades of increasingly visible rural drug use and its related harms [2, 3]. While once restricted to southern California, methamphetamine has had its largest impacts in rural states such as Oklahoma, Iowa, and Missouri [4]. More recently, Nebraska has joined this list, with increasing evidence of following in its neighbor’s footsteps. The most recent Centers for Disease Control and Prevention data for Nebraska reveal that substance abuse treatment rates for prescription opiate use in 2010 was seven times what it was a decade earlier, and opiate-related overdose deaths in Nebraska are rapidly approaching the number of deaths due to automobile accidents [5]. In neighboring Missouri, overdose deaths have exceeded automobile deaths for several years [5].

Understanding and preventing health-related harms arising from drug use is complex [6, 7]. Years of addiction studies point to multifactorial causes for drug abuse, ranging from altered neurological function [8], behavioral factors [9, 10] and psychosocial determinants [11], all operating in complex feedback loops. Complicating this are the physical, social, and emotional effects of blood born infections such as HIV, hepatitis B and C and tuberculosis, as well as a longer list of sexually transmitted infections frequently contracted in the context of drug use [12]. Although many human-system/virus-system interactions are now understood [13], how these are embedded in specific social contexts often remains unknown [14].

The few studies of rural drug use that exist show marked differences in rural versus urban drug users across demographic variables such as age or gender [15, 16] and large discrepancies in both the contexts of drug use and patterns of drug-related health consequences [17–19]. Other less direct disparities include differing social stressors, lower overall health levels and health care access, a dearth of substance use treatment facilities [20], unstable incomes, sparse social networks, and continuing high levels of social stigma around drug use and its related infections [21]. All of these mark rural drug use as vastly different from use in urban areas. Despite a small number of important exceptions [15, 18, 20], the urgent challenge of understanding drug use in rural settings is significantly under-examined.

### The Problem

The United States’ relationship with drugs is woven into our national history [22], from tobacco and rum to coffee and stimulant-laced drinks, to the use of performance enhancing drugs in our national pastime. Further, moral, health, economic, and psychological questions around drugs have been continually raised and disputed. This consistency does not reflect stasis, however. The nature and challenge of drug use continues to change rapidly [23], evolving in location and time in reaction to enforcement and education that would curtail use and driven forward by consumer demand and high profits [24]. Because much of this demand (and profit) is rooted in personal addiction, debates about drug use invoke individual and ethical dimensions not associated with other health issues. Recent findings on rising rates of white male mortality, in part due to the increasing numbers of drug-related deaths, has once again raised the question of America’s relationship with substance abuse [25] and drugs as they reflect or contribute to national moral decline [26–30].

Beneath the public furor, however, the health sciences have made considerable progress in understanding the etiology of drug use and in uncovering the link between drug use and its myriad associated harms. To name only a few areas of progress, the last three decades have seen remarkable advances in the neurochemistry of drug effects, the virology of pathogens whose spread is often rooted in drug use [13], the understanding of social determinants of drug initiation [31] and clinical approaches to drug cessation. Researchers now speak openly and optimistically about an HIV vaccine and a cure for AIDS. Safer and more reliable forms of opiate inhibitors are emerging every year and as the National Institute on Drug Abuse (NIDA) points out, drug treatment effectiveness is now on par with the success rates of treatments for diabetes and asthma [32].

However, this promising scientific news has not always translated to better health outcomes. An outbreak of HIV infection in southern Indiana in 2015 revealed surprisingly widespread rural drug injection [33]. Current estimates of this outbreak are that 40 percent of the local network of people who inject drugs were infected in less than four months, a scenario not witnessed in urban areas since the early 1980s [33, 34]. The risks go beyond HIV and Clark County, Indiana, where the majority of cases were located. A medical state of emergency was declared in neighboring Madison County based on a high prevalence of hepatitis C virus (HCV) infection discovered in the process of understanding the HIV outbreak [34].

Nationally, overdose-related deaths and rates of opiate addiction in the United States are once again near record highs [35], a situation that Department of Health and Human Services (DHHS) Assistant Secretary Richard Frank recently identified as a top priority for the Administration and DHHS as a whole [36]. Even progress in slowing the use of cocaine cannot mask the immense growth in the use of other stimulants such as methamphetamine [37]. Today, the diversion of prescription and over-the-counter medicines into the illicit drug market is more prominent than at any time since regulation began [38].

These issues are exacerbated by changes in use patterns that have seen a dramatic increase in the use of illicit drugs in rural and sub-urban areas [39]—a change that (perhaps relatedly) locates problems of addiction and drug-related harms in those regions where overall health care infrastructure is struggling to remain viable. As a result, and in ways not seen before, rural drug use has come to urgent national attention. Data on rural drug use and its harms justify this attention. Methamphetamine use in Nebraska, Oklahoma, Iowa, and Missouri now rivals any region in the US [4]. Substance abuse treatment needs in rural states dwarf available services [5], and overdose rates in rural states in the Central Plains exceed 30 deaths per 10,000 residents in some rural counties [40]. Arrest data for cocaine, methamphetamine, or heroin possession in rural counties in the region are similar to urban zones with similar patterns of drug use such as Maricopa County Arizona, and Dallas, Texas [41].

Comparisons of rural to urban drug use within Nebraska show that rural users start using drugs at a younger age, are more likely to use and sell methamphetamine, and use non-marijuana illicit drugs at a higher rate than their urban counterparts [16, 42]. Their methods of use are more risky as well. Between 40 and 50 percent of rural methamphetamine users in Nebraska prefer injection-based use, nearly twice the urban rate and a similar ratio was found for lifetime rates of methamphetamine-related psychosis [16, 43]. Daily count methadone use in Nebraska in 2012 was four times the rate of 2008 and overall estimates suggest that only 8.6 percent of illicit drug-dependent individuals received treatment during 2012 [43]. In all, 3,594 Nebraskans and 8,131 Iowans were admitted for treatment for cocaine, methamphetamine, and opiate abuse in 2013, with methamphetamine by far the most common.

Like rural Indiana prior to the 2015 outbreak, rates of HIV are low in Nebraska, Iowa, and Kansas. However, risk is high. All of these states have highly restrictive syringe access laws [44–46] and high treatment deficits [47, 48]. The current treatment need-to-capacity ratio for opioid addiction in Nebraska is nearly 6:1, ranking third worst in the United States behind Arkansas and South Dakota (each roughly 7:1) and well behind treatment capacity in Connecticut, Massachusetts, and New York, where ratios are roughly 1:1 [49]. Together, these factors foster significant vulnerability to outbreaks like that seen in Indiana and in other rural states [50]. While little surveillance information is available for rural areas in the Central Plains, data from regions with similar use patterns suggest HCV rates of 40 to 50 percent among rural injectors [51–53]. This marks both a hidden precursor to the HIV risk made possible by current drug use patterns and a serious health crisis in the making.

To address this issue, we need to recognize that clear differences exist for rural drug use that make most urban intervention programs ineffective. These differences include: 1) the settings in which drug use takes place (i.e., social, moral, economic, geographical, and environmental contexts); 2) the patterns of use and demographics of rural drug users; and 3) the conditions and capacities for treatment, intervention, and general care.

### Rural Contexts and Drug Use

Elevated levels of behavioral risk for residents of rural areas has been recognized among youth [54] and adults [39, 55], although it is also widely recognized that data on rural risk remain uneven and insufficient [56]. Efforts to understand geographical differences as they influence drug-related risks point to a range of “social ecological” factors underlying these differences [57, 58]. For example, according to Keyes and colleagues [59], the concentration of opiate abuse in rural areas is tied to general structural factors that differentiate rural areas from cities, including: 1) higher rates of opioid prescription, 2) youth outmigration, 3) larger kinship networks that facilitate informal drug trafficking, and 4) more economic stress.

Other approaches note macro-level social structural factors (e.g. marked differences in prisoner re-entry outcomes) for rural areas [60] or differences in rural drug policing [61]. Still others point to radically different racial [62, 63] and economic dynamics [64] related to rural use and risk patterns—factors that require different treatment and intervention strategies. However, not all research takes the urban/rural divide as paramount. Research on rural methamphetamine use points to the importance of local social contexts in creating micro-locational differences within rural areas [65], and research on rural cocaine use finds significant population level differences between users of different stimulants and their respective risk profiles [62]. Some recent evidence even looks at genetic-environment interactions and how these affect substance use susceptibility in rural areas [66, 67]. These approaches could also shed light on rural concentrations of drug use from genetic ‘founder effects.’ Such findings point to an important consideration—that the causes and implications of rural drug use are highly variegated and may be as different from one region to another, or even one county to another, as they are from generalized urban trends.

### Rural Patterns of Drug Use and Demographics of Rural Users

When we shift the focus to rural drug users themselves, there is again evidence of systematic differences between urban and rural areas, including who uses drugs and how [23, 68]. In a wide ranging series of studies in Appalachia, Havens and colleagues found marked differences between rural and urban drug users (mainly users of prescription opiates) in transitions from first use to first injection [17], patterns of initiation of opioids and polysubstance use [15, 69], gender propensities [70], and HCV infection [71], even while patterns of non-fatal overdose were similar [72]. Others have found significant rural/urban differences for methamphetamine users [65]. In Nebraska, Kansas, and Iowa, treatment admissions for methamphetamine are nearly equal for men and women [73], a startling contrast with other drugs and other regions. According to the Treatment Episodes Data Set (TEDS 2013), in Nebraska, one third of drug treatment admissions were for stimulants, and amphetamines were second only to alcohol in total treatment admissions [47]. Grant and colleagues found that rural methamphetamine users in the state were nearly twice as likely to only inject as their urban counterparts were (37.2 percent versus 20.2 percent) [16]. This risk profile implies radically different potential for HIV and related harms should the virus enter these communities. Other harms have already taken place: in the same report, Grant and colleagues found that lifetime prevalence for meth-related psychosis was much higher for rural (44.9 percent) than urban (28.7 percent) individuals, despite similarities across all other mental health areas [74].

Research on factors influencing user differences within rural areas has focused on many of the same issues that differentiate use among urban populations. Meyers found that adolescent risk and support factors for stimulant users differed along racial lines [75, 76], while Pope found differences for this group were (also) patterned by gender [77]. In both cases, similarities between urban and rural drug users were clear. These results point to an important conclusion—rural users require special consideration for multiple reasons, including decidedly higher rates of methamphetamine use, a higher proportion of users preferring injection, and a user population that is younger and riskier than urban counterparts [51, 78]. This conclusion holds true regardless of whether greater differences exist between urban and rural drug use, or within rural areas themselves,

### Rural Capacities for Treatment, Intervention, and Care

Several of the above issues contribute to low treatment success rates in rural areas. In looking at differences within rural areas, Oser and Harp discovered that cultural differences between home and treatment venues played a large role in rural treatment outcomes [79], findings echoed by McMaster for female methamphetamine users [80]. Jackson and Shannon, on the other hand, noted few differences in treatment seeking attitudes in rural versus urban pregnant women [81]. Rather, in their view, and in the view of others, these outcomes for rural treatment seekers may simply reflect a shrinking rural health care infrastructure [21, 64] and decades of general mortality differences across the full continuum of rural settings [82]. There are reasons to suspect this is the case, including a documented lack of available substance use disorder treatment facilities [83] and drug education programs [84] in rural counties. However, these may be only part of the problem, as the treatment needs caused by drug use often go beyond the actual user, affecting family, community, and environment [85]. These findings point to rural conditions that require specific attention to regional issues and differences within rural drug using populations. At the same time, we must keep in mind the clear evidence that larger structural features distinguish rural from urban zones, including differences in care.

In the United States, drug use and its associated health risks have traditionally been considered an urban problem. For example, the Centers for Disease Control and Prevention (CDC)’s National HIV Behavioral Surveillance (NHBS) Program on Injection Drug Use focuses entirely on the nation’s 25 largest urban areas, performing extensive surveillance testing among drug using populations in each of these cities every three years. In contrast, drug-related disease surveillance in non-urban zones is largely restricted to local law enforcement programs. In many rural areas, non-prison surveillance is non-existent. In the past, this emphasis on urban drug use has been justified by low rates of drug-related health impacts in non-urban zones. However, the evidence of the last five years shows that this is no longer the case.

## Discussion

Rural injection drug use and its related harms have come to national attention at the same time that drug use in the United States has undergone a radical reformulation. New clusters of prescription opiate users (23), significant numbers of whom transition to heroin and the near ubiquity of methamphetamine abuse across rural parts of the country [86] have transformed the national health landscape. Both of these phenomena have significantly impacted the Central Plains, a region one recent author refers to as “Methland” (22) for the sheer scope of use and for its intense impact in a quickly changing rural economic landscape. The harms associated with the use of these drugs goes beyond the more well-known viruses, with implications for sexually transmitted infections contracted in the context of drug use [87], mental health problems [88], and other social and family harms that ripple out from personal centers of addiction [89] (Table 1).

Despite timely and pressing challenges related to rural drug use, there are crucial limitations in the current state of research surrounding this issue. These challenges must be addressed if we hope to impact rural drug use. In general, the need for reliable physical, contextual and cognitive data related to the onset and desistance of drug use (and behaviors associated with drug use harms) is particularly critical for research on rural illicit drugs users [90]. Indeed, a significant amount of what we know about drug use in urban areas is based on successful, long-term cohort studies [90, 91], some of which continue today such as ALIVE [92], MIX [93] and VIDUS [94]. However, no rural equivalent currently exists and past rural studies have focused mainly on Appalachia [18].

When we look at the 32 counties in eastern Nebraska, western Iowa, and northern Kansas, we get a sense of the immensity of the challenge. By scaling up [95–97] drug-related (non-marijuana) arrest data from the Department of Justice's Uniform Crime Reporting Program (UCRP; [77]) and hospitalization data from the Substance Abuse and Mental Health Services Administration (SAMHSA; [78]), we can estimate the size of the drug using population in these counties to be ~20,000 individuals (which is ~2.7 percent of the counties’ population of ~ 740,000). This figure is commensurate with national prevalence estimates of drug use as a percentage of total population [79].

While research is needed, immediate solutions to the health risks posed by rural drug use are available. Modern day harm reduction points to three basic means for minimizing the personal and social risks associated with drug use, especially injection drug use preventative strategy. This startlingly contradictory position was underwritten by entrenched (and essentially irrational) opinions about syringe exchange programs—namely, the idea that making clean needles available in exchange for dirty ones somehow encourages drug use among those not already using drugs, or that it increases the level of drug use among those already using. This has been shown repeatedly and uniformly to be false in both cases [98]. To be clear, there is no evidence in three decades of research to suggest either assumption, yet it governs policy in nearly half of the states in the US, and nearly all states in the Central Plains. This is clearly one of area of public health where rational, data-driven outcomes have yet to be adopted in public policy arenas, and thus an area where immediate progress could be made that would save lives and lower treatment costs, even while it protects the wider non-using public from potentially dangerous outbreaks of HIV or hepatitis C. Temporarily closing the barn door long after the horse has left, as was done in Indiana, is unlikely to prevent HIV and hepatitis C outbreaks in places like Nebraska, Missouri, Kansas, Oklahoma or Iowa and much of the Central and Northern Plains [99].

### Opiate substitution treatment

Treatment of opiate addiction has included opiate substitution for more than five decades. Among the earlier methods was methadone treatment, an opiate that produces less of a “high” that is used in measured and medically supervised program lessen the effects of heroin or opiate withdrawal. Its effectiveness was debated for years, but recent retrospective reviews have found that methadone substitution treatment was highly effective not just in treating addiction, but in lowering on-going risk for infection during the treatment period and beyond [100].

New treatments using substitution drugs such as buprenorphine have proven even more effective than methadone, and have longer lasting success rates. Buprenorphine is a partial opiate agonist, meaning that it bonds to neurological receptors in the brain in a way that is similar to opium (or heroin or a range of opiate-based prescription pain killers), but does not produce the same effects as opiates (i.e., the opiate delirium experienced as a “high”) [100]. It is thus highly effective in staving off withdrawal symptoms without fulfilling the psychological need for escape.

By lessening withdrawal effects, buprenorphine allows the user to gradually reduce use and “ease off” of the drug, normally while receiving addiction therapy aimed at understanding and lessening the causes of psychological addiction. By allowing the latter (psychological addiction) to be treated without the experience of withdrawal (physiological addiction), the user stands a greater chance of successfully confronting those issues that inspired drug use/initiation in the past.

Despite these successes, buprenorphine treatment is very rare in the Midwest and Central Plains. Because treatment takes place in a doctor’s office and under medical supervision, the availability of treatment is dependent on sufficient numbers of doctors who are willing to participate. Arkansas, North Dakota and Nebraska rank worst in the US in terms of the ratio of opiate addicts to buprenorphine prescribing doctors. This is partly due to the scope of the opiate addiction problem in these states, and partly due to lack of rural medical infrastructure. In some ways, the lack of buprenorphine prescribing doctors reflects the general lack of medical services in rural areas, but in the situation is exaggerated by the stigma attached to drug use and addiction. This is particularly troubling because in these states, opiate addiction is increasingly a rural problem.

This too is a solvable problem, but one that currently lacks political attention. By allowing and promoting buprenorphine services in existing rural facilities, successful treatment for growing opiate addiction in the region could be expanded dramatically in the Central Plains. Given that the treatment is covered by virtually all public and private forms of insurance, the cost to these states, after the initial training and implementation, would be minimal—and the savings in lost productivity time, emergency room visits, and general public risk for drug use related diseases would be substantial.

### Naloxone

Where buprenorphine is a partial opiate agonist, naloxone (often known under the commercial name of Narcan) is an opiate antagonist. This means that it operates by blocking the attachments of opium to neurological receptors. In effect, naloxone prevents opium from having an effect on the brain. For this reason, it is often used in emergency situations to reverse an overdose.

As discussed above, drug overdose rates in the Central Plains have grown dramatically over the last decade. Missouri, Oklahoma and Wyoming have rates of overdose nearly double the rates of New York, California, Texas, or Virginia and nearly double the rates of Eastern rural states that have receive considerably more public attention such as Vermont. Notably, some states in the Central Plains still do not make naloxone available to the public, nor have they passed “Good Samaritan” laws protecting bystanders who report overdose incidents to emergency services, or who administer naloxone to someone who has overdosed.

Unlike syringe exchange or buprenorphine availability, considerable progress has been made in this area, however. Central Plains states with high levels of overdose have taken action to make overdose deaths less likely by making naloxone more available and its use in an emergency more protected. Holdouts continue, though, including Missouri, Kansas, South Dakota, Iowa, Minnesota and Wyoming. This is difficult to understand, other than to point out the overall punitive attitude toward addicts in these states. Again, there now exist easy and low cost means to mitigate the relationship between drug use and overdose death, held back only by a seeming desire to punish addicts for their addiction.

Taken together, these three inexpensive and cost effective programs could greatly reduce the disease risk of injection drug use, facilitate addiction recovery, and reverse the rising rate of accidental death associated with overdose. All of these programs have been employed in other regions with considerable success, and with none of the feared side-effects of increased drug use. The lesson that we need to recognize in Nebraska and the Central Plains is that data driven means for dealing with rising rural drug use in our region are available, and acting only after the problem can no longer be ignored ensures that these means will cost more and be more widely needed. In short, the time to act is now.

## Figures and Tables

**Table 1:** The scope of illicil drug use in 32 contagious countries of eastern Nebraska, Western lowa and northern Kansas.

Number of countries	32
Population of countries (P)	740,705
Arrests related to drugs (UCRP)	978
ER admissions for drug use (SAMHSA)	3.050
Est. drug user pop. size (5% arrest rate)	19,560
Est. drug user pop. size (15% ER rate)	20,333
Drug user pop. size as percentage of P	2.7%
